# Exercise-Induced Bronchospasm and Allergy

**DOI:** 10.3389/fped.2017.00131

**Published:** 2017-06-08

**Authors:** Serena Caggiano, Renato Cutrera, Antonio Di Marco, Attilio Turchetta

**Affiliations:** ^1^Respiratory Intermediate Care Unit, Pediatric Department, Bambino Gesù Children’s Hospital, Rome, Italy; ^2^Sleep and Long Term Ventilation Unit, Pediatric Department, Bambino Gesù Children’s Hospital, Rome, Italy; ^3^Sport Medicine Unit, Bambino Gesù Children’s Hospital, Rome, Italy

**Keywords:** exercise-induced bronchospasm, asthma, atopy, allergy, sport, children

## Abstract

Sport is an essential part of childhood, with precious and acknowledged positive health effects but the impact of exercise-induced bronchoconstriction (EIB) significantly reduces participation in physical activity. It is important to recognize EIB, differentiating EIB with or without asthma if the transient narrowing of the airways after exercise is associated with asthmatic symptoms or not, in the way to select the most appropriate treatment among the many treatment options available today. Therapy is prescribed based on symptoms severity but diagnosis of EIB is established by changes in lung function provoked by exercise evaluating by direct and indirect tests. Sometimes, in younger children it is difficult to obtain the registration of difference between the preexercise forced expiratory volume in the first second (FEV1) value and the lowest FEV1 value recorded within 30 min after exercise, defined as the gold standard, but interrupter resistance, in association with spirometry, has been showed to be a valid alternative in preschool age. Atopy is the main risk factor, as demonstrated by epidemiologic data showing that among the estimated pediatric population with EIB up to 40% of them have allergic rhinitis and 30% of these patients may develop adult asthma, according with atopic march. Adopting the right treatment and prevention, selecting sports with no marked hyperventilation and excessive cooling of the airways, children with EIB can be able to take part in physical activity like all others.

## Introduction

As known physical activity is fundamental for growth and long-term development in children, it has been shown to induce positive physiological and psychological effects, an improvement in cardiovascular, respiratory, and muscular systems. Furthermore, children undertaking physical training frequently modify their diet, with a reduced risk of overweight and obesity; in so doing, physical inactivity is considered to be an independent risk factor for various chronic diseases of adulthood (1). The terms “exercise-induced asthma” (EIA) and “exercise-induced bronchoconstriction” (EIB) are often used interchangeably. A consensus between the American Academy of Allergy, Asthma and Immunology, the American College of Allergy, Asthma and Immunology, and the Joint Council of Allergy, Asthma and Immunology used the term “EIB with asthma” for EIB with clinical symptoms of asthma and “EIB without asthma” for an acute airflow obstruction without asthma symptoms (2). A joint Task force of European Academy of Allergy and Clinical Immunology and European Respiratory Society described EIB as the reduction in lung function happening after exercise, as observed in exercise test, while defined EIA as symptoms of asthma occurring after exercise (3). In fact EIB is a temporary contraction of respiratory muscle after exercise that happens frequently in subjects without diagnosis of asthma, especially athletes, changing with the intensity of exercise and the environment (4). EIB is a distinct form of airway hyperresponsiveness, which is defined as the tendency of airways to constrict more easily and more forcefully than normal airways in response to a wide variety of bronchoconstrictor stimuli (5). Asthmatic subjects without anti-inflammatory treatment are at risk to have an asthma attack induced by exercise, up to 75–80% (6), but even people with no diagnosis of asthma may develop reduction in lung function after exercise, possibly such as a significant risk factor for the development of asthma (7). Consequently, EIB can occur in the presence or absence of asthma (4) and even if normally the physiologic response to exercise typically products in bronchodilation (8), subjects without asthma diagnosis may suffer from EIB.

## Prevalence

The prevalence of EIB ranges from 5 to 20% in the general population to even 100% in people with uncontrolled asthma. This huge variability depends not only on the criteria used for diagnosis, because there is not a gold standard (4), but also on the population samples studied. EIB is, in fact, reported to be particularly frequent (up to 45%) in children (9). It has been observed that exercise-induced wheezing in <5 years in childhood has been reported to be a strong predictor of persistent asthma in adulthood (10). Children and adolescents are more frequently affected than adults (11) and in the Oslo birth cohort study “Environment Childhood Study” 36.7% of 10-year-old children with a diagnosis of asthma showed EIB, with a positive exercise test, while 8.6% had a positive EIB test in the entire population-based birth cohort (12). An estimated 12% of the pediatric population has EIB and 30% of these patients may develop adult asthma (13). EIB reflective of the underlying asthma condition is reported in up to 90% of subjects with identified asthma but EIB can occur in individuals without a clinical history of asthma (14). Athletes particularly suffer from EIB without known asthma (15, 16), more frequently those with risk factors such as lung injury secondary to prematurity (17) or neonatal chronic lung conditions (18). However EIB is more commonly reported when asthma is associated to heavy exercise like in competitive athletes. Recent data in literature describe a prevalence of 10% of EIB in school children (19), according with results reported in two studies 15 years apart, estimating EIB from 17% (20) to 7.7% (21) or in 15% of a large pediatric study (22). But these assessments vary depending on the population and method of diagnosis in fact by using a free running test with peak flow monitoring the prevalence in one primary school population was 7.4% (23). In a pediatric Algerian population EIB prevalence has been described in up to 45% (24), up to 40% of individuals with allergic rhinitis. In fact among various risk factors for EIB is needed to identify history of allergy. The risk factors affecting the prevalence of EIB are multifactorial, including the presence and severity of asthma, family history of asthma or atopy including inhalant allergy, age, ethnicity, sex status, intensity and duration of exercise, presence of respiratory infection, atmospheric, and economic conditions, including an urban or rural setting and poverty (25, 26).

## Mechanism of EIB

Physical exercise is one of many non-pharmacologic and non-immunologic stimuli that can produce episodes of airway obstruction in patients with asthma. In asthmatic subjects the immune environment is tilted toward the Th2 side of the T helper cell axis as, for example, severity correlates IL-3, -4, -5, -9, and -13. Levels of IL-4 mRNA and protein are higher in asthmatic airway cells and its activation of STAT6 results in airway hyperresponsiveness, mucin production and goblet cell hyperplasia. IL-4 and IL-13 increase the number of NKT cells in the airways, increase B-cell IgE production and IL-13 has been implicated in airway remodeling (27, 28). While the number of mast cells and eosinophils is abnormally high in people with currently active asthma, mast cells are found in high density in healthy non-asthmatic subjects (29). The mediators include prostaglandins and histamine that contribute to the onset and severity of the EIB, and cysteinyl leukotrienes that sustain the presence of EIB and retard recovery of forced expiratory volume in the first second (FEV1). Sensory nerves are also likely to be involved, in that they respond both to a change in osmolarity and to cysteinyl leukotrienes (30). Physical activity is the second leading cause of acute airway obstruction and ranks only behind viral upper respiratory tract infections in this regard (14). Classical mechanisms behind EIA and EIB include the so-called osmolar (or airway drying) and vascular (or “thermal”) hypothesis (31). Both hypotheses are based on the marked increased ventilation during physical activity, leading to increased water and heat loss through respiration. Increased water loss increases the osmolality of the extracellular fluid lining the bronchial mucosa, causing water to move extracellularly possible through the water channels, aquaporins, and bronchial epithelial cells to “shrink,” with an increase in intracellular ion concentration (17) and release of inflammatory mediators from mast cells, eosinophils, neutrophils, and other inflammatory cells including newly formed eicosanoids (32, 33). The epithelium may serve as a key regulator of the balance of eicosanoids in the airways by activating the release of bronchoconstrictive eicosanoids in inflammatory cells in close contact and by alterations that reduce the synthesis of the protective PGE2 (34). So the main factor is now thought to be the inflammation induced by changes in airway osmolarity, and both osmolar and thermal mechanisms may work together under conditions of significant heat loss involving airway rewarming after cooling of the airways as the initiating mechanism. During normal tidal breathing, the nose functions like a rebreathing organ with warming up and humidifying the inspired air. The respiratory heat loss increases with increasing exercise intensity due to the increased ventilation. If the inhaled air is cold, the respiratory heat loss with the resulting cooling of the airways is further enhanced (35, 36). The cooling of the airways results in reflex parasympathetic nerve stimulation causing bronchoconstriction through the vagal nerve (37). At first, it is notable that a reflex vasoconstriction of bronchial venules to conserve heat occurs, but when exercise ends, the increased ventilation ceases, as does the cooling stimulus, causing a rebound vasodilatation of the peribronchial venules. The resulting smooth muscle constriction due to nerve stimulation and mucosal edema due to vasodilatation in susceptible individuals reduces the size of the bronchial lumen with increased airways resistance (37). Even in bronchoalveolar lavage fluid, it is possible to find increased peptidoleukotriene concentrations because of the bronchial epithelial damage with eosinophil and neutrophil influx due to exercise (29).

## Atopy and Environmental Factors

Environmental factors such as temperature of inhaled air, the humidity and intensity of exercise have a significant effect on the induction of bronchoconstriction. In a study made with the aim to understand if school also could be a significant site of allergen exposure for children in terms of environmental factors, such as atmospheric conditions and the presence of allergens, both potentially predictive of exercise-induced symptoms during physical education, it was showed an effect of environmental factors, like humidity and barometric pressure, and environmental allergens, in particular it was observed of cat allergens, on the occurrence of the EIB and cough in schoolchildren (38). Atopy and upper airway diseases are also known to influence EIB. A study, made on adult population, mean age 22.8 years, with the objective to identify differences between EIB alone and EIB with asthma, did not reported significant difference in total IgE, atopy rate, and house dust mite sensitization rate but it was observed an increased sensitization rate to outdoor molds in EIB-positive patients (39). Evidence supports airway hyperresponsiveness and decreased lung function from chronic exposure to air pollutants during exercise. The increased development of atopic dermatitis (AD) in infancy and subsequent allergic rhinitis and asthma in later childhood is known as the atopic march (40). The progressive atopy is dependent on various underlying factors such as the presence of filaggrin mutations as well as the time of onset and severity of AD. A dysfunctional skin barrier was suggested as a site for allergic sensitization to antigens and colonization of bacterial super antigens. This induces systemic Th2 immunity that predisposes patients to allergic nasal responses and promotes airway hyper reactivity. AD is a major risk factor for the development of asthma, and children with AD have an increased odds ratio of developing asthma compared to children without AD in several longitudinal studies. The main risk factors for progression and persistence of asthma are IgE sensitization and early onset and severity of AD. Epidemiologic studies illustrate strong associations between rhinitis and asthma (41). Studies on the prevalence of asthma in patients with rhinitis vary considerably, but it has been reported to be as high as 80% (41). Many patients with allergic rhinitis have lower airway hyperreactivity or bronchial hyperresponsiveness. Allergic rhinitis as a risk factor for developing asthma has been supported by several studies (42). Ciprandi et al. (43) showed that nasal symptoms, airflow, and markers of inflammation (eosinophils, Th2 cytokine levels) directly correlate with lower airway markers including FEV1. It was found that approximately 75% of subjects with asthma report rhinitis; patients with rhinitis have increased risk for asthma and lower airway reactivity compared to patients without rhinitis (44); furthermore it was observed that risk for asthma increases from 2.0% in subjects without rhinitis to 18.8% in subjects with allergic rhinitis when exposed to either pollen or animal dander. Exercise may trigger allergic respiratory, systemic and skin disorders (1).

## Symptoms

A large variety of symptoms could be present: shortness of breath, enhanced breathing effort, chest tightness, cough, wheezing, decreased performance, increased fatigue, chest pain, and chest tightness (18). Exertional dyspnea in children may be a presenting symptom but rarely in isolation as this usually is suggestive of an alternate diagnosis of deconditioning or vocal cord dysfunction (VCD) or cardiopulmonary disorders. Symptoms that are more apparent on inspiration may indicate exercise-related laryngeal obstruction (45). VCD occurs during exercise, while EIB usually occurs afterward but may overlap, and the two entities may coexist with VCD and asthma. In the majority of studied children with asthma, the time to maximal bronchoconstriction after exercise is short, suggesting that the onset of EIB occurs during exercise (46). Symptoms typically appear within a few minutes after the start of exercise and may continue for 10 or 15 min after the end of workout. Anyone can experience these symptoms, especially if out of shape, but with EIB, they are more severe than would be considered normal (47, 48). Even non-specific symptoms of stomachache or sore throat in children may be indications of EIB (49).

## Diagnosis

**Figure 1:**
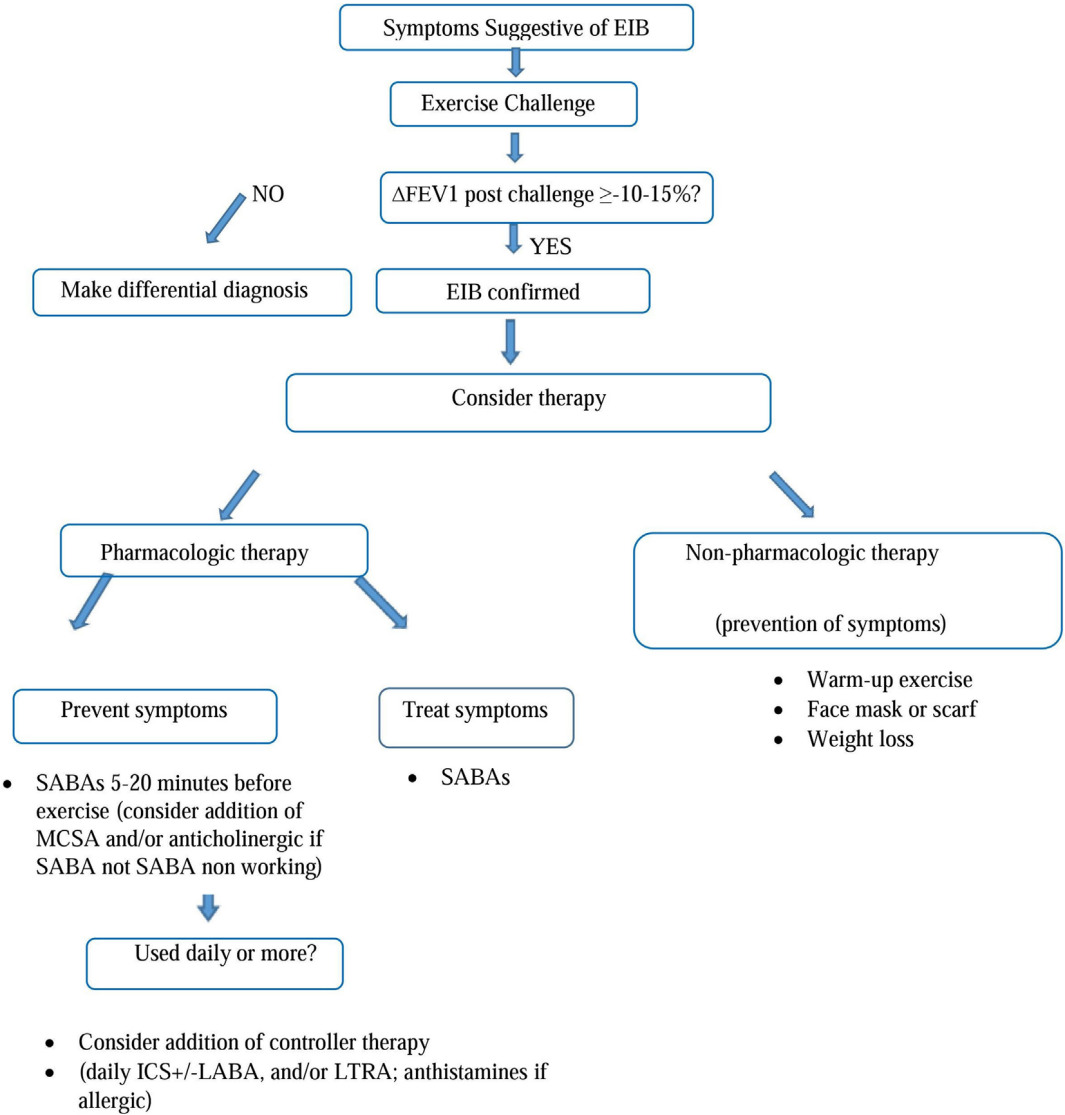
Algorithm for diagnosis and treatment of EIB. [Figure adapted by author from Parsons et al. (22). This reference provides the most contemporary state-of-the-art American Thoracic Society (ATS) guidelines on EIB]. Abbreviations: EIB, exercise-induced bronchoconstriction; FEV1, forced expiratory volume in the first second; SABA, short-acting b2-agonists; MCSA, mast cell stabilizing agent; ICS, inhaled corticosteroids; LABA, long-acting b2-agonists; LTRA, leukotriene receptor antagonists.

The diagnosis of EIB is established by changes in lung function provoked by exercise, not on the basis of symptoms neither on therapeutic trials without diagnosis (4). In the clinical practice, often an exercise challenge can be indicated in subjects with suggestive symptoms but showing to have normal to near-normal spirometry both before and after bronchodilator (4). This challenge can occur with free running, treadmill running or cycling, or with exercise surrogates such as mannitol (18), particularly in patient with known asthma for reason of safety (4). The objective tests of bronchial responsiveness are divided into “direct tests” [methacholine (MCH), histamine] and “indirect tests” [exercise, mannitol, adenosine 5-monophosphate, non-isotonic aerosols, and hyperpnea (EVH)]. The MCH test is widely used. MCH acts as an analog of acetylcholine, directly stimulating the cholinergic receptors in the airways’ smooth muscle. It has a high sensitivity but a low specificity for active asthma and a low sensitivity to recognize EIB (29, 50). Mannitol test can reproduce the “osmolar” mechanism of EIA/EIB, through the osmotic action of this agent, demonstrating sensitivity and specificity comparable to the MCH for the diagnosis of EIA/EIB (29, 51, 52). Another effective test, made by ventilating dry air with CO_2_ for 6 min through a low-resistance circuit at a rate higher than that usually realized during maximum exercise, is the EVH test (19). Test is positive when a ≥10% sustained reduction in FEV1 is achieved. However, at the moment specialists prefer to detect serial lung function measurements after a specific exercise to identify EIB and to quantify the severity of the disorder. Normally the value assessed is the FEV1 because more reproducible and more selective than peak expiratory flow rate. The airway response is reported as the percent fall in FEV1 from the baseline value. The exercise challenge requires pulmonary function monitoring. The ideal setting should be characterized by dry air preferably with relative humidity <20% and temperature <22°C. Anyway at the moment, we there is not a firm consensus for the conditions under which exercise should be performed (4). The challenge is performed with 2 min to ramp up to at least 85% of the maximum heart rate or 95% for children or athletes, maintaining this heart rate for 6 min (53) and monitoring pulmonary functions every 5 min until 30 min after challenge (15, 19, 29). The difference between the preexercise FEV1 value and the lowest FEV1 value recorded within 30 min after exercise is expressed as a percentage of the preexercise value. EIB diagnosis requires a ≥10%fall in FEV1 within 30 min of challenge (Figure 1). The severity of EIB can be graded as mild, moderate, or severe if the percentage fall in FEV1 from the preexercise level is >10% but, 25%, >25% but, 50%, and >50%, respectively (22). The surrogates for exercise testing, developed because easier to implement than exercise challenge, such as eucapnic voluntary hyperpnea or hyperventilation, hyperosmolar aerosols, including 4.5% saline, and dry powder mannitol (22) were established before the widespread use of inhaled corticosteroids (ICSs). Today, a decline in the FEV1 of ≥30% in a person taking inhaled steroids is considered severe EIB. Exercise challenge testing induces high levels of ventilation ideally by a rapid increase in exercise intensity over 2 to 4 min. Most protocols involve running while breathing dry air (10 mg H_2_O/L) with a nose clip in place. A valid test need the achievement of an optimal ventilation, greater than 21 times the resting FEV1, for 4 to 6 min of exercise, after which serial measurements of lung function are executed. Alternative diagnostic tests comprise inhalation of hyperosmolar saline, eucapnic voluntary hyperpnea of dry air and inhalation of dry powder mannitol (54). Is possible to diagnose EIB in 3–6 years of age children by measurement of FEV0.5 and airway resistance using the interrupt technique in 5–12 years old (15, 19, 29). In fact among bronchoprovocation tests in preschool children, interrupter resistance (RINT) has been used in association with spirometry to evaluate the presence of EIB. 36% of children (18 of 50) showed the presence of broncho-obstruction after exercise, showing that RINT may be a valid alternative method, definitely easier to run than spirometric examination, in these cases (55). Differential diagnosis is needed with other respiratory diseases (Table 1), distinguishing inspiratory stridor alone from inspiratory stridor with or without expiratory wheezing (4). For example is essential to differentiate EIB from such as the exercise-induced VCD, that should be consider when the respiratory distress is inspiratory and it occurs with inspiratory respiratory stridor during maximum exercise. This illness may co-occur with VCD. To differentiate EIB/EIA from other lung illnesses is essential to prescribe the best therapy as asthma treatment has no effect upon this and others pneumological diseases (1).

**Table 1:** Exercise-induced asthma: differential diagnosis [modified from Del Giacco et al. (1)].

Diagnosis	Patients	Symptoms	Test
Exercise-induced asthma	Children, asthmatic or not, during physical activities	Dyspnea, wheezing, cough, thoracic pain	Spirometry before and after exercise, refer to a sport medicine specialist
Exercise-induced vocal cord dysfunction	Asthmatic children and children active in sports	Symptoms occur during maximum effort. Symptoms disappear when exercise is stopped unless the patient continues to hyperventilate. The dyspnea is of inspiratory type. There are audible inspiratory sounds from the laryngeal area and no signs of bronchial obstruction No effect of pretreatment with inhaled bronchodilator	Exercise test with maximal exercise load, 6–8 min duration Direct laryngoscopy during exercise test
Exercise-induced hyperventilation	Children and adolescent active in sports, children in general	Hyperventilation with respiratory dyspnea and increased end-tidal CO_2_	Case history, observation during dyspnea
Exercise-induced anaphylaxis	Children and adolescent active in sports	Shortness of breath accompanied by pruritus, urticarial and low blood pressure	Allergy skin test, identify possible dietary triggers
Chronic lung diseases	Children affected by difficult to treat asthma, cystic fibrosis etc	Exercise limitation due to reduced lung function	Maximal exercise stress test with oxygen consumption, lung function test

## Treatment

We should classify EIB treatment in pharmacological and non-pharmacological therapy. Non-pharmacological therapy includes maneuvers to improve air condition during exercise by pre warming and humidification, making warmup before the exercise, improving general physical conditioning such as weight loss if necessary (22). Preventing water loss by using a face mask may promote humidification and attenuate EIB (4). Nowadays need validation both dietary supplementation with omega-3 fatty acids and ascorbic acid and measures to reduce sodium intake appearing inconclusive in reducing EIB (4). Many therapeutic options are available to prevent EIB. For example, inhalation of β2 agonist at recommended dose immediately before exercise effectively prevents the symptoms by stimulating β2 receptors on mast cells inhibiting release of contractile mediators and inducing relaxation of the airway smooth muscle like an antagonist of such mediators (15). Principal pharmacologic treatments are short-acting b2-agonists (SABAs) and long-acting b2-agonists (LABAs), ICSs, and leukotriene receptor antagonists (LTRAs). Inhaled anticholinergic agents (ipratropium) and antihistamines furthermore should be useful to control symptoms. For patients with EIB, the official American Thoracic Society (ATS) clinical practice guideline suggests administration of an inhaled SABA before exercise (48). The SABA is typically administered 15 min before exercise. A controller agent is generally added whenever SABA therapy is used daily or more frequently. In patients with EIB who continue to have symptoms despite using an inhaled SABA before exercise, or who require an inhaled SABA daily or more frequently, because of serious side effects, ATS recommend against daily use of an inhaled LABA as single therapy. The potential duration of protection afforded by a β2 agonist is 4–6 h for SABAs, like albuterol or terbutaline, and twice as long for LABAs, such as formoterol or salmeterol. However, tolerance may be developed if these drugs are taken daily (56), with reduction of duration of protective effect approximately to 2 h for SABA and 6 for LABA. Probably the tolerance is due to the downregulation of β2 receptors on the mast cell with poor efficacy in preventing mediator release. Furthermore because of the tolerance EIB may have a more rapid onset and an incomplete and slow answer to a SABA (4, 57). ATS recommendations provide administration of ICS not only before the exercise but daily. In fact ICS should be taken for 2–4 weeks to see the maximal improvements. ICSs alone or in combination with other drugs can improve the EIB control in terms of severity and symptoms frequency but they do not prevent the tolerance from LABA daily therapy (4). Even if the effective role of leukotriene antagonists are not defined, montelukast can be used daily or intermittently (4) to prevent EIB, as observed efficacy at 2, 12, and 24 h after a single oral dose of 10 mg (58). Montelukast is reported to reduce the% fall in FEV1 by 40–60% and to reduce the recovery’s time of FEV1, without tolerance induction (59). Combined therapy of antihistamine with montelukast can be useful and more effective than a single drug but the combination is not generally recommended (15). In terms of stabilization of mast cells, sodium cromoglycate and nedocromil sodium may be a valid therapeutic option, effective since from the first administration, made shortly before exercise, but with short duration of action both alone and with other drugs are indicated in case of EIB (4, 60). Inhaled anticholinergic agent before exercise may be considered as an alternative and suggested treatment. However, in the clinical practice, a daily inhaled ICS or daily LTRA appears at the moment as the first option, chosen case by case depending on patient’s lung function and characteristics. There is an intrapatient and interpatient variability in the treatment efficacy. In allergic patients, with poor improvements and control with only inhaled SABA before exercise, ATS suggests administration of an antihistamine. For all patients with EIB, interval or combination warm-up exercise before planned exercise is recommend and in subjects with EIB who exercise in cold weather, routine use of a device (i.e., mask) that warms and humidifies the air during exercise is recommended. In conclusion, any intervention that reduces the amount of water lost or increases the water content of the inspired air will reduce the severity of EIB. EIB is often the first sign of asthma to come and the last to go with treatment so that control of EIB is an indicator of asthma control (15).

## Importance of Sport in Children with EIB

The impact of EIB significantly reduces quality of life and participation in sports. When participating in systematic physical training, the asthmatic adolescent or child improves fitness and quality of life as confirmed by a Cochrane-based meta-analysis of eight training studies, including 226 asthmatics from 6 years of age (30). Physical activity is generally accepted to be an advantage to young children in terms of bone development, motor skills, improved cardiovascular fitness, and self-esteem (61). There is evidence that asthmatic children with well-controlled disease, even those with documented bronchial hyperresponsiveness, can achieve levels of exercise performance similar to those of non-asthmatics (62). Several studies have identified significant improvements in aerobic fitness (63, 64) and asthma-related benefits such as reduced hospital admissions, reduced absences from school, reduced medication use, and fewer doctor’s visits after exercise performance (65). Moreira et al. also demonstrated, in children with persistent allergic asthma, that a physical training program did not increase airways inflammation but decreased their total and allergen-specific IgE levels (29, 66). Finally, preliminary data show that regular exercise reduces IL-2 production, meaning that lymphocytes are probably less responsive to exogenous stimuli, and IL-4 producing lymphocytes are also reduced, suggesting a better clinical condition for allergic people that exercise regularly (67). Overall, children with asthma should be medicated appropriately and encouraged to participate in regular physical activity. Successful management allows for participation in the chosen sport for the pediatric recreational athlete as well as with elite Olympic athletes with asthma. Olympic athletes with asthma have won gold and silver medals proportionately with their non-asthmatic competitors (18). But which sports? Subjects who participate in endurance and winter sports as well as swimming are at higher risk for EIA/EIB. Long-duration exercise and very low air temperature easily expose these patients to the osmolar and vascular changes in the airway, fundamental in the EIA/EIB pathophysiology. Types of training and atopy are independent risk factors for EIA/EIB but combining the two factors in a logistic regression model and atopic speed, EIA/EIB is significantly more common in athletes compared with control subjects, especially in swimmers but also in long-distance runners and track and field athletes. Furthermore atopic disposition appears strongly associated with increased bronchial responsiveness, being the most important risk factor for EIB and asthma (68). However, the apparent highest prevalence of bronchial hyperresponsiveness in swimmers is due to the fact that athletes selected swimming as their primary event but is known that swimming does not cause EIA/EIB as much as other activities like running, for example, even by avoiding outdoor allergens (68). In addition, for the asthmatic athlete it is also important to avoid strenuous exercise during temporarily increased exposure to “biological stress.” This can be increased aeroallergen load, extreme cold air environment, or strenuous exercise too close to a recent viral respiratory tract infection. With an early and precise diagnosis, insightful precaution protecting the airways from extreme biological stress and an early start of anti-inflammatory treatment, the progression of bronchial hyperresponsiveness and asthma in these children having sport may usually be well controlled. It should be useful warm up with gentle exercises for about 15 min before to start more intense physical activity. Cover the mouth and nose with a scarf or face mask when subjects exercise in cold weather and try to breathe through the nose during the exercise. Sports that require only short bursts of activity are preferred, including volleyball, gymnastics, baseball, wrestling, golf, swimming, football and short-term track and field events. Some swimming events can demand constant activity, but the warmth and humidity from the water make it easier for people with EIB to breathe, activities such as walking, hiking, and recreational biking. Sports and activities most likely to cause EIB symptoms are those requiring constant activity or done in cold weather, such as soccer, basketball, long-distance running, ice hockey, ice skating, and cross-country skiing (47, 48). Sports with low risk for the development of asthma and bronchial hyperresponsiveness are the ones in which the physical effort is of short duration and in which high ventilatory levels are not reached. Medium-risk sports are team sports in general, in which the alternation of aerobic and anaerobic phases, as well as the relatively brief periods of continuous high-intensity exercise (in any case usually lower than 5–8 min), results in a lower risk of bronchial hyperreactivity. High-risk sports, as already stated, are endurance and winter sports in general (26). It is important that children’s physical activities are adapted to their situation, teachers should know what to do in emergency and that parents help their children to take their medication properly but the most important thing is to choose a sport that each children enjoys (69).

## Author Contributions

AT and RC conceptualized and designed the review. SC contributed to data collection, carried out the initial analyses and interpretation of data in literature, and wrote the manuscript. AT coordinated and supervised analysis and interpretation of data, critically reviewed, and revised the manuscript. AM contributed to data collection and interpretation of literature. All the authors approved the final manuscript as submitted and agreed to be accountable for all aspects of the work.

## Conflict of Interest Statement

The authors declare that the research was conducted in the absence of any commercial or financial relationships that could be construed as a potential conflict of interest.
